# Novel Compound Heterozygous CFAP53 Variants in a Fetus With Situs Inversus Totalis: A Case Report

**DOI:** 10.7759/cureus.103723

**Published:** 2026-02-16

**Authors:** Stylianos Lagios, Andreas E Spathi, Vassilis Papanikolaou, Theoni Leoutsakou, Theochari Eleni, Maria Kavvadia, Theano Stauroulaki, Anastasios Xefteris, Angeliki Malamidou, Angeliki Rouvali, Afroditi P Pegou, Athena P Souka, Marianna Chatziioannoy, Aspasia Destouni, Panagiotis Antsaklis, Andreas Pampanos

**Affiliations:** 1 Department of Genetics, Alexandra General Hospital, Athens, GRC; 2 Department of Genetics, Eurogenetica MSA, Athens, GRC; 3 First Department of Obstetrics and Gynecology, Alexandra General Hospital, National and Kapodistrian University of Athens, Athens, GRC; 4 Third Department of Obstetrics and Gynecology, School of Medicine, Aristotle University of Thessaloniki, Thessaloniki, GRC

**Keywords:** array cgh, cfap53, conventional karyotyping, heterotaxy, situs inversus, whole exome sequencing

## Abstract

Heterotaxy defects can include morphological deviations that affect left-right symmetry. In this case report, we observed novel compound heterozygous *CFAP53*variants that affect laterality. Whole exome sequencing (WES) was performed during pregnancy. Prenatal exome sequencing is particularly valuable for fetuses with laterality defects, given the increasingly high diagnostic success observed in this category of morphological abnormalities. Molecular testing plays an important role in genetic counselling, allowing parents to make decisions about pregnancy, calculating risk, and often predicting possible medical problems due to the nature of the disease.

## Introduction

Situs solitus (SS) refers to the asymmetric position that organs occupy within the left-right (LR) axis of the body. Any laterality defects can give rise to medical problems, especially in the heart [1,2]. Several genes have a role in maintaining correct laterality in embryos, in particular the coiled-coil domain-containing (*CCDC*) protein. This is supported because mutations in genes *CCDC39*, *CCDC151, *and *CCDC40 *give rise to situs inversus totalis (SIT) and primary ciliary dyskinesia (PCD) [3,4].

SIT is a rare congenital anomaly defined by a complete mirror-image transposition of the thoracic and abdominal viscera. In this condition, the heart is located on the right side of the thorax (dextrocardia), with associated reversal of the liver, spleen, stomach, and intestinal anatomy [5]. The estimated incidence ranges from one in 10,000 to one in 20,000 live births, and inheritance is most commonly autosomal recessive, often related to defects in LR embryonic axis determination [6].

Any laterality defects in fetuses prompt immediate genetic testing because patients with heterotaxy are known to suffer from chronic respiratory tract infections, male infertility, and an affected sense of smell due to the damaged cilia and flagella [7]. There are several more syndromes related to heterotaxy such as an increase in complex congenital heart disease from 0.6% in normal anatomy to 3-9% in patients with SIT [8]. Around 20-40% of patients with LR symmetry defects show vascular anomalies such as interrupted inferior vena cava (INT-IVC) and preduodenal portal vein (PDPV) [9]. Hepatic anatomy can also be affected since patients with LR asymmetry demonstrate a higher frequency of hepatic arterial anatomy and biliary atresia [10,11]. Interestingly though, mutations on the *CFAP53 *gene do not cause ciliopathies but isolated SIT [12-14].

## Case presentation

**Figure 1:**
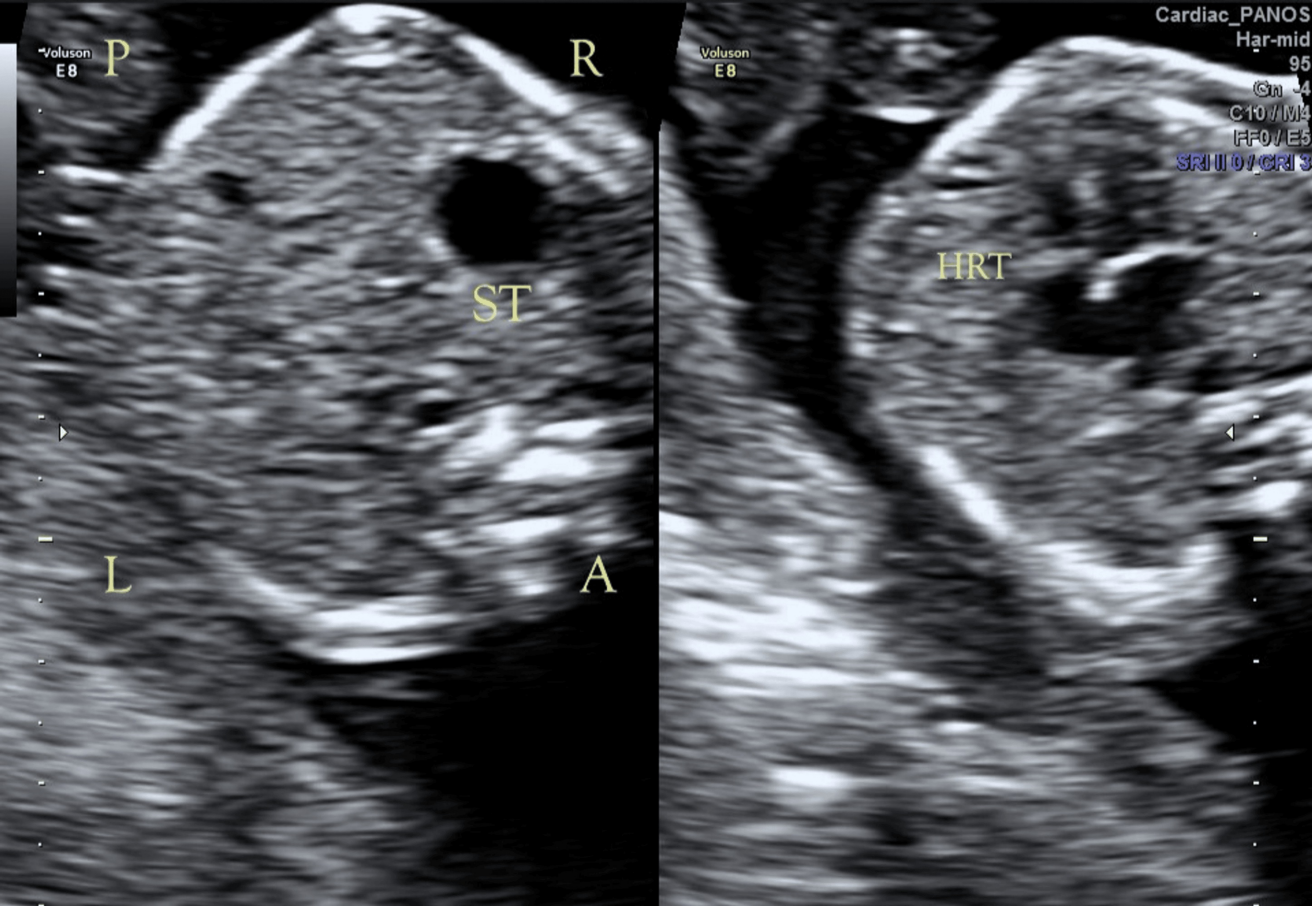
Transverse ultrasound images Transverse ultrasound images of the fetal abdomen (left) and thorax (right) obtained at 20 weeks and four days gestation. Orientation markers indicate fetal right (R), left (L), anterior (A), and posterior (P). The fetal stomach (ST) is visualized on the right side of the abdomen, and the fetal heart (HRT) is visualized with its apex directed toward the right thorax. These findings are suggestive of a right-sided visceral and cardiac orientation (right-sided stomach and heart).

A woman was referred at 20+4 weeks of pregnancy because of ultrasound findings indicating SIT without additional anomalies (Figure 1). The large intestine was visualized to the right of the midline. Echocardiogram of the fetus showed no pathological findings except dextrocardia. The couple reported no history of any Mendelian disorder. Amniocentesis, conventional karyotyping (R-bands by heat using Giemsa (RHG) banding), array comparative genomic hybridization (aCGH), and whole exome sequencing (WES) were performed. DNA was extracted using the Promega 16 Maxwell extractor (Madison, WI, USA), and aCGH analysis was performed using the aCGH GenetiSure Cyto 8×60k (Agilent Technologies, Santa Clara, CA, USA), according to the manufacturer's instructions. Slides were scanned with SureScan microarray, and analysis was performed with the Agilent 5.1.2.1 CytoGenomics analysis program. For WES, the exons, adjacent intronic sequences (+/- 5 nucleotides), and selected regulatory and distant intronic sequences on 18,433 human genes were sequenced. For this purpose, a library of target regions was created (Ion AmpliSeq™ Exome RDY Kit, Thermo Fisher Scientific, Waltham, MA, USA) and sequenced on the Ion GeneStudio S5 Prime System (Thermo Fisher Scientific). Bioinformatic analysis was performed using validated algorithms through the analysis system VarSome Clinical (Saphetor SA, Lausanne, Switzerland). About 98.5% of the target regions were sequenced to a depth of 50×. The sequences were mapped to the human reference genome GRCh37/hg19. The nomenclature of the alleles detected follows the latest version of the Human Genome Variation Society.

No findings were observed by conventional karyotyping or aCGH, while WES analysis revealed a compound heterozygosity of the *CFAP53* gene. The first variant was a frameshift deletion, referred to as c.870del, and the second was a change at a splice site, referred to as c.473+1G>A. According to the American College of Medical Genetics and Genomics (ACMG) criteria, the two findings are predicted as likely pathogenic (Table 1). The identified *CFAP53* variants were independently validated by bidirectional Sanger sequencing. Genomic DNA extracted from the amniotic fluid was amplified by polymerase chain reaction (PCR) using primers flanking the variant-containing regions. PCR products were purified and subjected to cycle sequencing using dye-terminator chemistry. Sequencing fragments were resolved by capillary electrophoresis on an ABI 3500 Genetic Analyzer (Thermo Fisher Scientific). Sequence traces were analyzed by alignment to the reference sequence, and both variants were considered confirmed based on concordant forward and reverse reads with high signal quality. Each parent was heterozygous for one of the two variants, with one parent carrying the frameshift variant and the other carrying the splice-site variant, confirming compound heterozygosity in trans in the fetus.

**Table 1:** Primary findings The first variant is at position 93 of the fifth exon of transcript NM_145020.5, located on the negative strand of chromosome 18q21.1. It is a frameshift deletion of one nucleotide at the 290th amino acid. The second variant is at position 1 of the third intron of transcript NM_145020.5, located on the negative strand of chromosome 18q21.1. HGVS: Human Genome Variation Society; SNV: single nucleotide variant; ACMG: American College of Medical Genetics and Genomics

Mutation	Transcript	Gene(s)	Exon	Variant type	Zygosity	Coverage	Allelic balance	ACMG rules
HGVS protein: A291Pfs*6 p.(Ala291ProfsTer6); HGVS coding: c.870del	NM_145020.5	CFAP53	Exon 5 of 8 position 93 of 219	Deletion (1)	Heterozygous	102	0.48	PVS1,PM2 _Supporting
HGVS protein: p.?; HGVS coding: c.473+1G>A	NM_145020.5	CFAP53	Intron 3 of 7 position 1 of 9279	SNV	Heterozygous	96	0.45	PVS1,PM2 _Supporting

## Discussion

SIT is a congenital condition in which thoracoabdominal organs are arranged as a mirror image of their normal positions. This results from the disruption of LR axis formation during early embryogenesis, a process mediated by motile and sensory cilia at the embryonic node [15]. Mutations in ciliary and centrosomal genes such as *CCDC39*, *CCDC151, *and *CCDC40 *have been implicated in abnormal organ laterality [3,4]. The present case identifies a compound heterozygote fetus with SIT. The two novel *CFAP53 *variants, c.870del and c.473+1G>A, expand the mutational spectrum of the gene and further illustrate the diagnostic value of prenatal WES.

*CFAP53 *encodes a coiled-coil protein involved in the structural organization of motile cilia and flagella. Functional studies in *CFAP53*-knockout mice demonstrate SIT, hydrocephalus, and male infertility which is consistent with a role in microtubule assembly at the ciliary basal body. Unlike mutations in genes causing PCD, *CFAP53* defects appear to selectively affect nodal cilia function during embryogenesis without impairing respiratory cilia [16].

In this report, the two novel *CFAP53 *variants are predicted to lead to the production of a truncated protein and a splicing error, respectively [17]. Both variants are rare or absent in the Genome Aggregation Database (gnomAD) and ClinVar and are characterized as likely pathogenic by the ACMG criteria.

While SIT is present in approximately half of PCD cases, *CFAP53-*related laterality defects differ fundamentally from classical PCD. Individuals with *CFAP53 *mutations often lack chronic respiratory symptoms, sinusitis or bronchiectasis, which are typical features of PCD [18]. This clinical distinction reflects the specific involvement of embryonic nodal cilia in *CFAP53*-associated disease rather than the dysfunction of airway cilia.

Identification of *CFAP53 *variants in this case enabled a precise molecular diagnosis during pregnancy. Given the autosomal recessive inheritance pattern, the recurrence risk for future pregnancies is 25%. Thus, the importance of WES in prenatal diagnostic and reproductive planning is of utmost importance [19,20].

From a clinical perspective, identifying *CFAP53 *variants underscores the relevance of integrating genetic analysis into prenatal imaging findings. Establishing a molecular diagnosis informs prognosis, facilitates family counseling, and guides postnatal management. From a research standpoint, expanding variant databases and performing functional validation in cellular and animal models remain crucial to accurately interpret novel *CFAP53 *variants. Future investigations should also explore possible modifier genes that influence phenotypic variability in laterality disorders.

## Conclusions

Any clinical indication of SIT or heterotaxy should prompt genetic testing, as pathogenic variants are identified in a significant proportion of cases in genes associated with laterality disorders and other ciliary or LR patterning genes.

This is the first report to our knowledge to demonstrate the implication of two novel and uncharacterized variants to SIT. The case adds to growing evidence that *CFAP53 *mutations contribute to isolated laterality defects. The discovery of novel compound heterozygous *CFAP53 *variants supports the gene's role in LR symmetry and highlights the value of prenatal WES for diagnosing and understanding laterality disorders. In silico predictions and functional studies would have strengthened the assessment of variant pathogenicity in this case; however, these analyses could not be performed. Ongoing collection of genotype-phenotype data and functional validation will be essential to elucidate the precise mechanism by which *CFAP53 *influences embryonic asymmetry.
